# Multiple Sclerosis and the Blood-Central Nervous System Barrier

**DOI:** 10.1155/2013/530356

**Published:** 2013-01-15

**Authors:** Alan M. Palmer

**Affiliations:** MS Therapeutics Ltd., Beechey House, 87 Church Street, Crowthorne, Berks RG45 7AW, UK

## Abstract

The central nervous system (CNS) is isolated from the blood system by a physical barrier that contains efflux transporters and catabolic enzymes. This blood-CNS barrier (BCNSB) plays a pivotal role in the pathophysiology of multiple sclerosis (MS). It binds and anchors activated leukocytes to permit their movement across the BCNSB and into the CNS. Once there, these immune cells target particular self-epitopes and initiate a cascade of neuroinflammation, which leads to the breakdown of the BCNSB and the formation of perivascular plaques, one of the hallmarks of MS. Immunomodulatory drugs for MS are either biologics or small molecules, with only the latter having the capacity to cross the BCNSB and thus have a propensity to cause CNS side effects. However, BCNSB penetration is a desirable feature of MS drugs that have molecular targets within the CNS. These are nabiximols and dalfampridine, which target cannabinoid receptors and potassium channels, respectively. Vascular cell adhesion molecule-1, present on endothelial cells of the BCNSB, also serves as a drug discovery target since it interacts with **α**4-**β**1-integrin on leucocytes. The MS drug natalizumab, a humanized monoclonal antibody against **α**4-**β**1-integrin, blocks this interaction and thus reduces the movement of immune cells into the CNS. This paper further elaborates on the role of the BCNSB in the pathophysiology and pharmacotherapy of MS.

## 1. Introduction

Multiple sclerosis (MS) is an acquired autoimmune disease that affects both the brain and the spinal cord leading to a variety of symptoms, including changes in motor function, sense perception, and mental function, along with fatigue [1–3]. The disease presents in different forms that follow distinct patterns of evolution and rates of disability progression [4]. The most common form is relapsing-remitting MS (RRMS), which affects about 85% of people with MS (pwMS), is more common in females than males (by a ratio of 2 to 1), and has an average age at diagnosis of 29 years [5]. RRMS is characterized by acute attacks (relapses) followed by partial or full recovery (remission) and contrasts with primary progressive MS (PPMS), which affects about 10–15% of pwMS, is diagnosed (on average) at age 40, has no gender bias, and is characterized by a steady and irreversible progression of functional impairments [6–8]. These two forms of MS also differ in their onset as PPMS begins insidiously, whereas the harbinger of RRMS is usually a transient impairment in motor or sensory function, together with white matter abnormalities shown by magnetic resonance imaging (MRI), but with insufficient evidence for a definitive diagnosis of MS [9, 10]. This is referred to as clinically isolated syndrome and, in most cases (80% after 20 years), progresses to RRMS [11]. At the other end of the RRMS continuum is secondary progressive MS (SPMS). The majority of those with RRMS convert to SPMS within two or three decades. It has a similar progressive and irreversible course to that of PPMS [12–14]. A final subset, which resembles RRMS, is progressive relapsing MS, which affects less than 5% of pwMS. It is characterized by a steady decline in neurologic function and clear superimposed exacerbations [4].

MRI plays an increasingly important role in the diagnosis and management of MS. It is also routinely used as the primary endpoint in proof-of-concept clinical trials evaluating potential new drugs for MS, and as the secondary endpoint in definitive phase III trials [15]. MRI scans distinguish fat from water. In T_1_-weighted images water is darker and fat brighter, whereas the opposite is the case in T_2_-weighted scans. Since myelin is predominantly lipid (and thus hydrophobic), areas of demyelination hold more water and so show up as either a bright white spot (in a T_2_-weighted scan) or a darkened area (in a T_1_-weighted scan). The sensitivity of the T_1_-weighted scans is often enhanced by the use of contrast agents, such as gadolinium diethylenetriaminepentaacetic acid. Gadolinium enhancement permits direct visualization of breaches in the BCNSB that accompany acute MS and so is used to visualize the number of new plaques in the CNS (usually the brain) of pwMS. It therefore provides a good measure of disease activity and helps distinguish between acute (or active) plaques and chronic (or nonactive) lesions [16]. In the brain, MS plaques are commonly round or ovoid and range in size from a few mm to more than 1 cm and are often found in the brainstem, cerebellum, and periventricular white matter [17–19].

Plaques are regions of demyelination and neuronal loss. They occur as a consequence of the movement of activated immune cells from the bloodstream into the brain or spinal cord (or both) across the blood-CNS barrier (BCNSB) [20]. The cascade of inflammatory change is probably initiated by autoreactive T-cells, particularly CD4^+^ and CD8^+^ T helper (Th) cells. CD4^+^ cells recognize antigens that are presented by major histocompatibility complex (MHC) molecules on specialized antigen-presenting cells and present them to specific T cell receptors [21]. The activation of immune cells by an autoantigen leads the CD4^+^ cells to commence pathological destruction of cells of self, particularly myelinated CNS neurons. The most prominent candidates for autoantigens are proteins present in myelin, such as myelin basic protein, myelin oligodendroglial glycoprotein, neurofascin and proteolipid protein. Other candidates include stress proteins such as B crystallin, which is found in the myelin sheath after activation via the inflammatory response [22, 23].

The cause of MS is not yet understood, but it is known that dozens of genetic variations act in concert with environmental factors to trigger disease pathogenesis. Evidence indicating the influence of the environment on gene expression is growing rapidly [24]. The relative contribution of nature and nurture to disease pathogenesis is provided by studies of genetically identical twins showing MS concordance rates of only 30% [25]. A series of recent genomic studies have confirmed a central role of the immune system in the pathogenesis of MS, with the MHC class II association now mapped to the HLA-DRB5∗0101-HLA-DRB1∗1501-HLA-DQA1∗0102-HLA-DQB1∗0602 extended haplotype [26–29]. Currently, the major environmental factors associated with MS are the Epstein-Barr virus (EBV) seropositivity, cigarette smoking, and low plasma concentrations of vitamin D3 [20, 30–33].

## 2. The Blood-CNS Barrier

The blood-CNS barrier (BCNSB) is a dynamic and complex cellular system that separates the CNS from the bloodstream. It does this by strictly controlling the exchange of both cells and molecules between the two compartments [34–36]. The largest surface area for exchange is the blood-brain barrier (BBB), which separates the bloodstream and the brain. Its sister barrier, the blood-spinal cord barrier (BSCB), separates the bloodstream and the spinal cord. There is also an epithelial cell barrier separating the bloodstream and the cerebrospinal fluid (CSF) at the choroid plexus and the arachnoid villi. Both the BBB and the BSCB comprise the endothelial cells of CNS blood vessels, along with a thick basement membrane and astrocytes. They display a unique phenotype characterized by the presence of endothelial cells that are connected by an intercellular adhesion complex. This forms the close contact between the adjacent cells known as tight junctions. This barrier function of the BCNSB is further enhanced by the relative paucity of fenestrae and pinocytotic vesicles. Collectively, this results in a low level of endocytosis and transcytosis, which severely restricts the movement of molecules and cells into the CNS via the transcellular route [34]. 

The BCNSB has two further barrier elements: (i) a metabolic barrier that contains a complex array of enzymes (including acetylcholinesterase, alkaline phosphatase, *γ*-glutamyl transpeptidase, and monoamine oxidases) that degrade different chemical compounds thus altering their pharmacological activity and (ii) a transport barrier that contains a variety of efflux transporters, including P-glycoprotein and breast cancer resistance protein [34, 37, 38].

Tight junctions are the critical component of the BCNSB as they control paracellular diffusion and maintain the structural and functional polarity of the specialized endothelial cells of the BBB and BSCB. Thus, the BCNSB contributes to the homeostasis of the parenchyma of the brain and spinal cord and provides protection against many toxic compounds and pathogens [34, 36, 39]. Indeed, the BCNSB is largely impermeable to compounds that are not lipophilic and have a molecular weight greater than 450 Da. This presents a major challenge for CNS drug discovery [40, 41]. 

## 3. The Role of the Immune System in MS

There are two general types of immune response: innate and adaptive. The innate system plays a role in both the initiation and the progression of MS by influencing the effector function of T and B cells [42]. Thus, for example, through the activation of specific (mainly toll-like) receptors in an antigen nonspecific manner, dendritic cells become semi mature and induce regulatory T cells to produce inhibitory cytokines such as IL-10 or tumour necrosis factor-*γ*. As the dendritic cells mature, they polarize CD4^+^ T cells to differentiate into Th1, Th2, or Th17 phenotypes; it is the Th1 phenotype that promotes inflammation. 

The adaptive response is initiated by the presentation of a specific antigen to T lymphocytes by the antigen-presenting cells (APCs). These APCs include B cells, dendritic cells, microglia, and macrophages. Several types of T cells can be activated by APCs and initiate the adaptive immune response. The key T cells involved are Th1, Th2, and Th17. Th1 cells secrete proinflammatory cytokines (e.g., interferon-*γ* and IL-12), as do Th17 cells (IL-17, IL-21, IL-22, and IL-26). By contrast, Th2 cells secrete anti-inflammatory cytokines (e.g., IL-4 and IL-13). Regulatory T cells, another CD4^+^ T-cell type, regulate effector Th1, Th2, and Th17 cells. In addition to CD4^+^ T-cells, CD8^+^ T cells mediate the suppression of CD4^+^ T-cell proliferation through the secretion of perforin, which is cytotoxic to CD4^+^ T cells and thus leads to their inactivation [43, 44].

## 4. The BCNSB and the Immune System

It was once thought that the CNS was completely isolated from the immune system and so was “immunologically privileged.” However, it is now recognized that this separation is incomplete since immunological surveillance of the CNS has been shown to occur routinely; it also appears to vary with age and brain region. Thus, rather than regarding it as immunologically privileged, the CNS may more accurately be described as immunologically specialized [46, 47].

**Figure 1:**
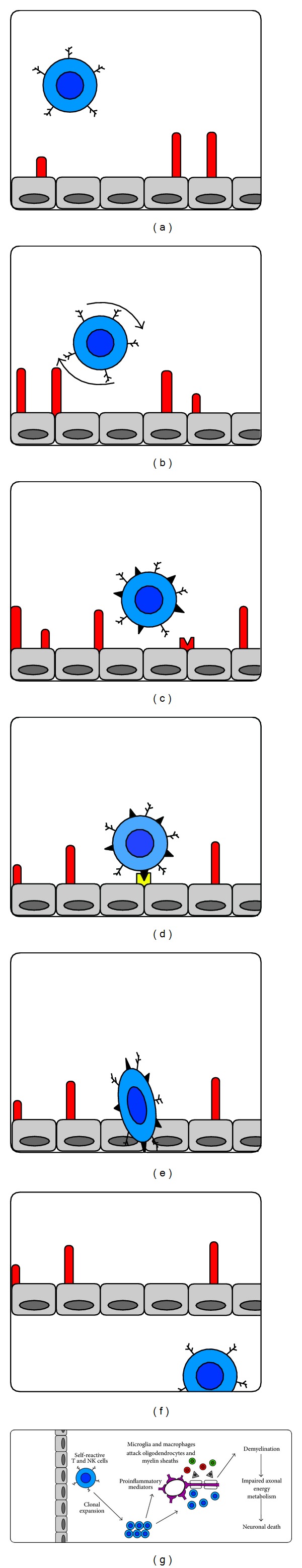
The role of cell adhesion molecules in the movement of activated T cells and natural killer cells across the blood-CNS barrier. (a) Tethering through the interaction of glycosylated PSGL-1 on leukocytes and P-selectin on endothelial cells. (b) Rolling of leukocytes along endothelial cells. (c) Integrin activation on leukocytes. (d) Firm adhesion through the interaction of *α*4*β*1-integrin and vascular cell adhesion molecule-1 expressed on the endothelial cell layer. (e) Paracellular movement of immune cells into CNS parenchyma (extravasation). (f) Presence of leukocytes in CNS parenchyma. (g) Once in CNS parenchyma, leukocytes increase in number by clonal expansion and then attack the entire supramolecular complex of myelin. This includes (i) a critical antibody response to various myelin proteins and lipids, (ii) initiation of the complement cascade and T and natural killer cell attack of certain key portions of various myelin antigens and (iii) release of cytokines, notably tumour necrosis factor, which stimulates macrophages, microglia and astrocytes, to produce nitric oxide [45].

The movement of immune cells from the bloodstream into CNS parenchyma occurs through a sequential and coordinated process involving (i) tethering, (ii) rolling, (iii) adhesion (binding), and (iv) extravasation across the BCNSB. This involves the binding of adhesion molecules with respective ligands (Figure 1). The capture and rolling of immune cells, such as leukocytes, is mediated by the selectin family of adhesion molecules and their sulfated, sialylated, and fucosylated glycoprotein ligands. Selectins exist in 3 forms: P, E, and L. L-selectin is localized on leucocytes, whereas E-selectin and P-selectin are found on the endothelium. Of these, the most efficient tethering molecules are P-selectin and L-selectin, with L-selectin playing a primary role in lymphoid tissues and P-selectin in other tissues. P-selectin is localized in the Weibel-Palade bodies of endothelial cells and *α*-granules of platelets. It is rapidly translocated to the cell surface in response to a variety of inflammatory stimuli such as oxidized lipoproteins, lipopolysaccharides, and thrombin. The main counter ligand is P-selectin glycoprotein ligand-1 (PSGL-1), a heavily glycosylated sialomucin expressed on most leukocytes. Binding takes place, under dynamic conditions to substantially slow leukocyte movement relative to mean blood flow, as these cells roll along the endothelium. *In vivo* studies using mice deficient in PSGL-1 have shown that PSGL-1 is the predominant, if not the only, P-selectin ligand expressed during inflammation. The function of PSGL-1 is closely linked to its posttranslational glycosylation, which is mediated by the Golgi enzyme core 2 b1,6-N-acetylglucosaminyltransferase (C2GnT). C2GnT is responsible for the synthesis of specific carbohydrate determinants on PSGL-1, including a 2,3-sialylated and a 1,3-fucosylated core 2 decorated O-glycans carrying the sialyl Lewis X (sLex) motif as a capping group [48–51]. 

The interaction between P-selectin and PSGL-1 thus leads to the capture of activated leukocytes onto the inside surface of blood vessels but is not sufficiently strong to fix leukocytes to the vessel wall. The anchoring of rolling leukocytes is achieved by the interaction between very late antigen-4 (VLA-4, *α*4*β*1-integrin) and vascular adhesion molecule (VCAM-1) [52]. Once anchored into position, leukocytes then move across (extravasate) the BCNSB through tiny spaces in the endothelium into CNS parenchyma [53, 54]. Normally, these cells then mount an attack on infectious agents within the CNS but, in the case of MS, they attack host cells [3, 35, 53, 55].

## 5. The Role of the BCNSB in the Pathophysiology of MS

The pathophysiology of MS is characterized by multifocal demyelination and neuronal loss, which probably occurs as a consequence of the movement of activated immune cells into the CNS. This requires passage through the BCNSB. Once leukocytes are in the CNS, they multiply by clonal and oligoclonal expansion, a process that is amplified by proinflammatory mediators (principally cytokines) through the recruitment of naive microglia and mediated by IFN*-*γ** and IL-12 [56, 57]. This leads to the principal pathological lesion of MS, the sclerotic plaque, which can be seen *postmortem* (by microscopy of stained tissue) and in the intact brain (by MRI imaging) [58–60]. Plaques grow slowly by radial expansion, as focal brain inflammation fades into diffuse parenchymal microglial activation and results in extensive abnormalities in normal appearing white matter [61]. With time, these plaques lead to the breakdown of the BCNSB, partly through the action of interleukins 17 and 22. BCNSB disruption permits the movement of more leukocytes into the CNS parenchyma, which then leads to multifocal perivascular infiltrates, predominantly T cells and macrophages [36, 62–64]. It is this movement of activated and committed leukocytes from the peripheral circulation through the BCNSB and into the CNS that is the most critical step in the formation of MS lesions. Once in the CNS, these cells propagate and trigger a sequelae of neuroinflammatory change that leads to the loss of both myelin and oligodendrocytes and culminates in neuronal loss by a mechanism that is not yet clear [65, 66].

Evidence indicating that the T lymphocytes specific for myelin antigens initiate an inflammatory reaction in the CNS is primarily derived from studies of allergic encephalomyelitis (EAE), a CD4^+^ T-cell-mediated animal (mainly rodent) model of MS. It involves inducing spinal cord inflammation by inoculation with human spinal cord [67–69]. 

There is good evidence to indicate that the BCNSB plays a pivotal role in the pathophysiology of MS from studies showing that, in EAE, antibodies against *α*4-*β*1-integrin, but not antibodies against numerous other adhesion receptors, prevented the accumulation of leukocytes in the CNS and the development of EAE [70]. Recognition of the significance of this data led directly to the initiation of human studies with a humanized monoclonal antibody to *α*4-*β*1-integrin (natalizumab) in pwMS [71]. In a recent systematic analysis of available data from multiple phase III clinical trials involving a total of 2,223 people with RRMS, natalizumab was found to reduce (i) the number of participants who experienced relapses, (ii) the number of individuals who progressed at 2 years, and (iii) MRI lesion activity [72]. Blocking the interaction between VLA-4 (*α*-4*β*1-integrin) and VCAM-1 therefore has therapeutic efficacy in both EAE and MS.

Blocking the interaction between PSGL-1 with its endothelial ligand P-selectin is another potential approach to MS pharmacotherapy [73]. However, this rationale has been questioned because it has been shown that anti-P-selectin antibodies, and PSGL-1 and P-selectin genetic deficiency, had no impact on the incidence, severity, or development of EAE [53, 73–76]. Nonetheless, the rationale for targeting PSGL-1 or P-selection is supported by data obtained from human tissues showing that (i) CD8^+^, but not CD4^+^, lymphocytes from pwMS displayed increased rolling on P-selectin using intravital microscopy, (ii) anti-PSGL-1 antibodies block the recruitment of CD8^+^ cells in brain vessels of pwMS [77]; (iii) increased numbers of circulating CD4^+^ T cells with high levels of PSGL-1 were found in pwMS patients, and (iv) these T cells had an enhanced ability to migrate across human brain endothelial cells *in vitro *[78]. In light of these human studies, it does seem that the interaction of selectins and PSGL-1 probably does play a key role in the pathology of MS. The failure of blocking the interaction between PSGL-1 and P-selectin to impact the development of EAE may well be attributable to the fact that EAE is mediated by CD4^+^ T cells. This is because CD8^+^, but not CD4^+^, lymphocytes from pwMS displayed increased rolling on P-selectin [77].

BCNSB disruption, which is partly mediated by CD8^+^ cells, permits the movement of more leukocytes into the CNS where they contribute to the loss of both myelin and oligodendrocytes and culminates in neuronal loss by mechanism that is not yet clear [3, 35, 43, 53, 55, 65, 66]. 

## 6. The BCNSB and MS Drugs and Drug Candidates

As shown in Table 1, most MS medicines are immunomodulatory agents, the first of which were *β*-interferon (IFN*β*) drugs. IFN*β*s are produced by expression in either Chinese hamster ovary cells (IFN*β*-1a) or in *Escherichia coli* (IFN*β*-1b). Other approved immunomodulatory drugs include glatiramer acetate (Copaxone), a random polymer of four amino acids (L-glutamic acid, L-lysine, L-alanine, and L-tyrosine) found in myelin basic protein, and natalizumab. All of the drugs are biologics and so have to be administered by injection. They are too large to cross the BCNSB since BCNSB permeation is restricted to compounds that are moderately lipophilic and with a molecular weight of less than 450 Da [41, 79]. 

The molecular target of IFN*β* drugs glatiramer acetate and natalizumab is in the circulating compartment (blood plasma and lymph fluid) and so BCNSB penetration is not required in order to achieve therapeutic efficacy. This makes MS unusual in the field of CNS medicines research as pharmacotherapy for CNS disorders normally requires BCNSB permeation [41]. The emergence of small molecule immunomodulatory drugs permits oral administration, which side-steps the difficulties associated with injectable biologics, including the generation of neutralizing antibodies and poor drug compliance [3]. However, compounds with a low molecular weight are much more likely to cross the BCNSB and interact with central neurons and possibly cause CNS side effects [41].

Natalizumab gained FDA approval in 2004 as a first-line treatment of pwMS with highly active RRMS and a second-line treatment for pwMS failing to respond to IFN*β* drugs. However, its human use was suspended in 2005 because of two reports of progressive multifocal leukoencephalopathy (PML). This is a severe and often fatal demyelinating disorder of the CNS caused by a lytic infection of oligodendrocytes by the JC virus and is characterized by progressive damage of white matter. Natalizumab was reintroduced in the United States, with a black-box warning of PML and approved in the European Union in 2006 after no additional cases of PML were identified in previously treated patients. The risk of developing PML is substantially reduced by (i) limiting treatment duration to two years (ii) excluding pwMS taking immunosuppressive drugs, and (iii) clinical vigilance, including demonstration of the absence of anti-JC virus antibodies in serum prior to the commencement of treatment [80, 81]. 

In two EAE models (C57BL/6 model, and a pertussis toxin-modified model in SJL/J mice), pretreatment with blocking antibodies to both *α*4-*β*1-integrin and P-selectin reduced firm adhesion of leukocytes to a similar extent; these antibodies also had greater efficacy when given together than when given separately. Despite evidence of blockade of leukocyte recruitment, no behavioral benefit was observed with either anti-selectin antibodies or genetic deletion of P-selectin in either EAE of the models. By contrast, antibodies to *α*4-*β*1-integrin delayed the behavioural onset of EAE. The time of onset of EAE was further delayed when *α*4-*β*1-integrin antibodies were combined with P-selectin antibodies. Combination treatment also reduced the severity of EAE [73]. This provides a compelling rationale for combining compounds that block the interaction between P-selectin and PSGL-1 with *α*4-*β*1-integrin blocking agents in order to improve the benefit/risk profile of monotherapy with such agents. This would include P-selectin-PSGL-1 blockers as an adjunct to both natalizumab and *α*4-*β*1-integrin blocking agents with a shorter half-life than natalizumab, such as firategrast (see below). 

Fingolimod (a structural analogue of sphingosine) was the first small molecule immunomodulatory MS drug to reach the market. Following its phosphorylation, it acts by mimicking sphingosine- 1-phosphate (S1P) and binds to S1P receptors on lymphocytes causing their downregulation. In the absence of S1P receptor signaling, CD4^+^, CD8^+^ T cells and B cells are unable to move from secondary lymphoid tissue, which substantially reduces the number of lymphocytes in the blood. Therefore, fewer activated leukocytes are available for movement into the CNS [82]. In people with RRMS, it has been shown to reduce (i) relapse rate, (ii) MRI lesions, (iii) brain-lesion activity, and (iv) loss of brain volume, as measured by MRI in comparisons with both placebo and IFN**β**-1a. It gained regulatory approval from the FDA in 2010 (as Gilenya) and the European Medicines Agency (EMA) the following year (as Gilenia) [3, 82]. Fingolimod crosses the BCNSB and therefore has the potential to interact with central neurons and cause CNS effects [83].

Teriflunomide (Aubagio) gained FDA and EMA regulatory approval for the treatment of RRMS in 2012. It is an active metabolite of the rheumatoid arthritis drug leflunomide and inhibits the mitochondrial enzyme dihydroorotate dehydrogenase and thus reduces pyrimidine synthesis. Because the production of activated T cells largely depends on *de novo* pyrimidine synthesis, pyrimidine depletion is thought to result in the inhibition of immune cell proliferation [84, 85]. On the basis of five phase III studies, teriflunomide appears to be efficacious with little evidence of serious adverse events [3]. However, it stays in the body for up to two years (distributed predominantly in the periphery) and may cause liver damage and birth defects. There is little evidence to indicate that teriflunomide crosses the BCNSB to enter the CNS [86]. 

There are a number of other low molecular weight compounds that are in phase III clinical trials or undergoing regulatory review for the treatment of RRMS. These are described below.

- Dimethyl fumarate (BG-12) is the methyl ester of fumaric acid, an intermediate in the tricarboxylic acid cycle. The mechanism by which therapeutic efficacy is achieved is not clear, although there is data to indicate that fumarate treatment induces IL-4-producing Th2 cells and generates type II dendritic cells that produce IL-10 instead of IL-12 and IL-23. Dimethyl fumarate is probably too hydrophilic to cross the BCNSB.
- Laquinimod is thought to act by shifting the immune response from Th1 to Th2. In experimental studies, it crosses the BCNSB and so has the potential to cause CNS side effects [3].
- Firategrast is a small molecule *α*4-*β*-integrin antagonist that has demonstrated efficacy on imaging endpoints in a phase II study of people with RRMS [87]. It has a molecular weight (MW) in excess of 450 Da (its MW is 499 Da) which is not compatible with good BCNSB penetration, but has other physicochemical properties (such as log P and the number of hydrogen donors and acceptors) that are consistent with BCNSB penetration [41]. Like natalizumab, firategrast indirectly targets the BCNSB by blocking the interaction between *α*4-*β*1-integrin on leukocytes and cell adhesion molecules on endothelial cells of the BCNSB. With firategrast, there is a reduced liability to cause PML as it has a much shorter half-life than natalizumab.

In addition to oral immunomodulatory drugs and drug candidates, two orally available medicines that treat specific symptoms of MS have recently entered the market. These are described below

- Ampyra, which is an extended release tablet containing dalfampridine. Dalfampridine is the broad spectrum potassium channel blocker 4-aminopyridine. It works by extending the action potential at both axons and nerve terminals, which leads to increased release of neurotransmitter and thus improves motor function in demyelinated or functionally impaired neurons. It gained approval on the basis of data from two phase III clinical trials that demonstrated that Ampyra (10 mg twice daily) improved walking speed (measured by the timed 25-foot walk) by an average of 25%. Though modest, this improvement was associated with a reduction in ambulatory disability in pwMS [88–90]. However, only one-third of the pwMS who received the drug were consistent responders.
- Dalfampridine has a poor therapeutic/risk ratio as potassium channels are intrinsic to normal function, particularly in the heart and the CNS. Thus, adverse events were mainly related to stimulatory effects on the nervous system. The most commonly reported side effects were MS relapse and epileptic seizures [89]. This is consistent with dalfampridine crossing the BBB, a conclusion supported by studies showing accumulation of dalfampridine in both the brain ISF and CSF compartments following systemic dosing in rats [91, 92]. Even so, an analysis of multiple published clinical studies indicates that adverse events are dose related, mild to moderate and transient, particularly at the low dose of 10 mg twice daily [93, 94].
- Nabiximols (Sativex) is a cannabis-based oral spray containing a defined quantity of specific cannabinoids, particularly tetrahydrocannabinol and cannabidiol, which are cannabinoid CB1 and CB2 receptor agonists, respectively [95, 96]. Since it acts on CNS neurons, BCNSB penetration is essential. Both tetrahydrocannabinol and cannabidiol were shown to readily penetrate the BBB in brain microdialysis studies of mice and rats [97]. Nabiximols was recently granted regulatory approval in a number of countries for the treatment of spasticity in MS. Reported side effects include dizziness and fatigue. They occur relatively frequently but are usually mild to moderate in intensity and rarely require drug discontinuation [98].

## 7. Conclusions

Unlike nearly all other blood vessels in the body, the endothelial cells of the BCNSB are bound together by tight junctions. This means that a neuroactive compound needs to take a transcellular route across the BCNSB in order to enter the CNS. These tight junctions, coupled with numerous efflux transporters and metabolizing enzymes, constitute a formidable barrier to the movement of both molecules and cells from the bloodstream into the CNS. The BCNSB plays a role in MS and its treatment at three levels.

- Pathophysiology. The movement of activated leukocytes across the BBB is a key event in the pathophysiology of MS. Once in the brain, these cells target epitopes on myelin, which initiates a cascade of neuroinflammation that leads to loss of myelin. This leads to BCNSB breakdown (which can be visualized by gadolinium-enhanced T_1_-weighted MRI scans) and the formation of plaques.
- Drug-induced pathophysiology. Natalizumab blocks immunological surveillance of the CNS, leaving the CNS immunocompromised. A detrimental consequence of this is the reactivation of the JC virus in the brain which can then lead to PML.
- MS pharmacotherapy. Most MS medicines are biological drugs and so their large size prevents their movement across the BCNSB. However, the emergence of small molecule immunomodulatory drugs will increase the probability of such compounds entering the CNS, which will increase the risk of CNS side effects [41]. Some MS drugs (such as nabiximols and dalfampridine) are centrally acting and so there is a requirement for them to cross the BCNSB in order to achieve the desired therapeutic effect.

IFN*β* drugs and glatiramer acetate have dominated the MS market for over a decade. This dominance is set to change with (i) the introduction of natalizumab, which targets the interaction between leukocytes and the BCNSB and has an impressive efficacy profile; (ii) the launch of three oral immunomodulatory drugs (fingolimod, dimethyl fumarate, and teriflunomide), with more (e.g., laquinimod and firategrast) in late stage development; (iii) a number of immunomodulatory monoclonal antibodies (alemtuzumab, daclizumab, and ocrelizumab) about to enter the market; and (iv) the emergence of drugs targeting symptom management, including motor dysfunction (dalfampridine) and spasticity (nabiximols). 

In conclusion, the BCNSB plays a pivotal role in both the pathophysiology of MS and MS pharmacotherapy. A deeper appreciation of this complex and dynamic barrier, particularly the endothelium of the cerebrovasculature, will provide a more complete understanding of the disease and its treatment.

## Figures and Tables

**Table 1:** Drugs approved for the treatment of multiple sclerosis [3].

Brand (and generic) name	Mechanism of action	Route of administration (dose)	Location of molecular target	Therapeutic efficacy
IFN*β*-1a (Avonex and Rebif)	Suppression of Th1 and enhancement of Th2 immune response	Avonex: once a week, i.m. (30 *μ*g). Rebif: three times a week, subcutaneous (44 *μ*g)	Circulating compartment^1^	Reduced relapse rate and MRI lesions
IFN*β*-1b (Betaseron and Extavia)	Suppression of Th1 and enhancement of Th2 immune response	Betaseron: every other day, subcutanous (250 *μ*g) Extavia: three times a week, subcutanous (250 *μ*g)	Circulating compartment	Reduced relapse rate and MRI lesions
Glatiramer acetate (Copaxone)	Tolerization with myelin-like antigens and modulation of autoreactive T cells	Every day, subcutanous (20 mg)	Circulating compartment	Reduced relapse rate and MRI lesions
Mitoxantrone (Novantrone)	Inhibition of the proliferation of T cells, B cells, and macrophages	Four times a year, intravenous. The lifetime cumulative dose is limited to 8–12 doses over 2-3 years (140 mg)	Circulating compartment	Reduced relapse and MRI lesions and disease progression
Natalizumab (Tysabri)	A humanized monoclonal antibody to *α*4-*β*1-integrin that prevents the movement of leukocytes from the bloodstream into the CNS	Every four weeks by intravenous infusion (300 mg)	Circulating compartment	Reduced relapse and MRI lesions and disease progression
Fingolimod (Gilenya/Gilenia)	Reduction in the number of lymphocytes in the blood by preventing their egress from lymph nodes through modulation of the sphingosine-1-phosphate receptor 1	Every day, oral (0.5 mg)	Circulating compartment	Reduced relapse rate and MRI lesions
Teriflunomide (Aubagio)	An immunomodulator with anti-inflammatory properties, probably through inhibition of dihydroorotate dehydrogenase	Every day, oral (7 or 14 mg)	Circulating compartment	Reduced relapse rate and MRI lesions
Dalfampridine (Ampyra)	Potassium channel blockade	Twice a day (10 mg)	CNS and PNS	Improved walking speed
Nabiximols (Sativex)	Cannabinoid CB1 and CB2 receptor agonism	Oromucosal spray (≤12 sprays per day)	CNS	Reduced spasticity

^1^Blood plasma and lymph fluid. BCNSB: blood-central nervous system barrier; CNS: central nervous system; PNS: peripheral nervous system; Th: T helper cell.
